# FAMeDB: A Curated Database for the Analysis of Fungal Aromatic Compound Metabolism

**DOI:** 10.34133/csbj.0049

**Published:** 2026-04-20

**Authors:** Tiago M. Martins, Rita C. Carmo, Cristina Silva Pereira

**Affiliations:** Instituto de Tecnologia Química e Biológica António Xavier, Universidade Nova de Lisboa (ITQB NOVA), Oeiras, Portugal.

## Abstract

Aromatic compounds represent the second most abundant class of organic molecules after carbohydrates, and their microbial metabolism is of broad relevance across multiple research disciplines. Metabolic pathways involving aromatic compounds span from highly conserved anabolic routes to more variable catabolic processes. Numerous peripheral catabolic pathways converge on a small number of central intermediates that undergo aromatic ring opening in the central pathways. Over the past decades, alongside numerous peripheral pathway genes, most of the catabolic genes constituting the central metabolic pathways have finally been characterized in fungi. Here, we present FAMeDB, a manually curated database of proteins involved in fungal aromatic compound metabolism, together with an R-based bioinformatic workflow for orthology data processing. The database currently includes 408 proteins, primarily enzymes, but also includes transcription factors and transporters. Entries span 82 species and 56 genera of fungi. Most entries are from Ascomycota (80%), with a substantial number from *Aspergilli* (44%). FAMeDB tested and validated the hypothesis that a cross-lineage fungal database can yield meaningful insights into aromatic compound catabolism. Application of FAMeDB and its tools enables the quick and accurate representation of aromatic metabolism across different fungal proteomes. This resource is designed to provide a useful and accessible platform for researchers worldwide, even those without specialized expertise in fungal aromatic catabolism, facilitating omics analysis and genomic comparisons.

## Graphical Abstract

**Figure:**
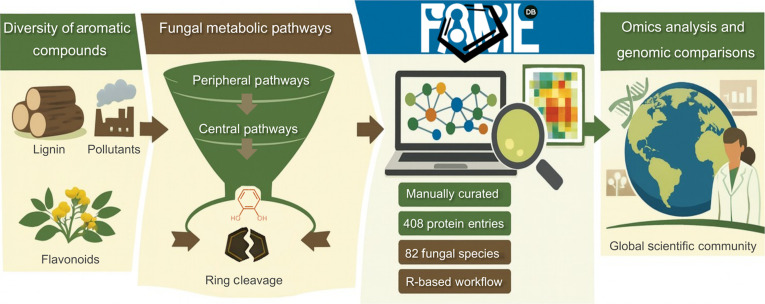

## Introduction

Aromatic compounds constitute the second most abundant class of organic molecules in nature, surpassed only by carbohydrates [1]. Their structural diversity and chemical stability underpin a wide array of biological functions and ecological interactions, making their metabolism a focal point across disciplines ranging from environmental microbiology to industrial biotechnology. Microbial degradation and transformation of aromatics are particularly critical for nutrient cycling, bioremediation, and the biosynthesis of high-value metabolites [2]. Despite extensive research in bacterial systems, fungal aromatic metabolism remains comparatively underexplored, even though fungi play pivotal roles in terrestrial ecosystems and industrial processes [2,3].

**Fig. 1.**
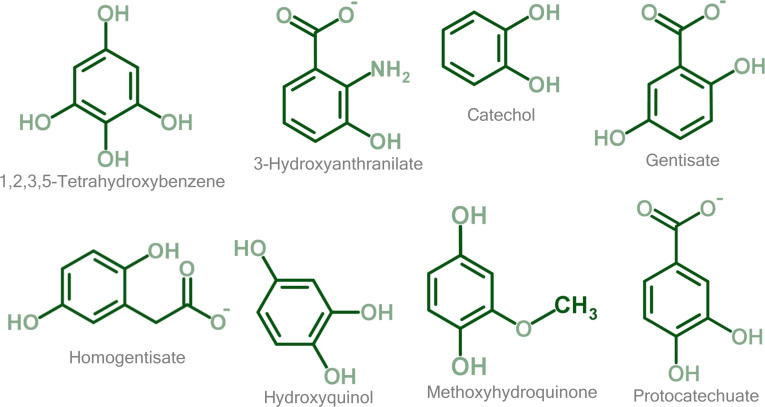
Fungal central ring-opening aromatic intermediates in the 8 central pathways of aromatic compound catabolism. International Union of Pure and Applied Chemistry (IUPAC) names listed left to right and top to bottom: benzene-1,2,3,5-tetrol; 2-amino-3-hydroxybenzoate; benzene-1,2-diol; 2,5-dihydroxybenzoate; 2,5-dihydroxyphenylacetate; benzene-1,2,4-triol; 2-methoxybenzene-1,4-diol; and 3,4-dihydroxybenzoate.

Fungi exhibit distinct metabolic strategies that set them apart from other microbial groups, particularly in their ability to process structurally complex aromatic compounds. Fungi contribute substantially to lignin degradation and the turnover of other plant-derived aromatic polymers and compounds (e.g., suberin and polyphenolic stilbenes), processes essential for carbon cycling and soil health [4–6]. Recent advances in fungal research in the omics era have revealed a wealth of previously uncharacterized genes and pathways involved in aromatic compound metabolism [2,7–10]. To date, 8 central ring-opening intermediates (Fig. 1) in the catabolism of aromatic compounds have been identified in fungi: 1,2,3,5-tetrahydroxybenzene, 3-hydroxyanthranilate, catechol, gentisate, homogentisate, hydroxyquinol, methoxyhydroquinone, and protocatechuate [2,7–10].

A mere 15 years ago, the genetic basis for only 1 of the 8 central pathways—homogentisate—had been described in fungi. Notably, both 1,2,3,5-tetrahydroxybenzene and methoxyhydroquinone were only lately characterized [10–12]. While the 1,2,3,5-tetrahydroxybenzene pathway currently seems restricted to fungi, an analogous methoxyhydroquinone pathway has been described in bacteria [13]. In the peripheral pathways, major advances have also been achieved in recent years, remarkably the elucidation of the cinnamic acids coenzyme A (CoA)-dependent beta-oxidative pathway and the characterization of multipurpose ortho-methoxyphenolases [14,15]. This surge in data, coupled with the increasing availability of fungal genomes, has opened new avenues for comparative and functional analyses, yet the lack of centralized, curated resources has hindered systematic exploration of fungal aromatic metabolism.

Existing resources relevant to the analysis of fungal aromatic compound catabolism include Carbohydrate-Active Enzymes database (CAZy) [16], Hydrocarbon Aerobic Degradation Enzymes and Genes (HADEG) [17], Kyoto Encyclopedia of Genes and Genomes (KEGG) [18], and BioCyc [19]. However, their utility for investigating fungal metabolic pathways involving aromatic compounds remains limited. For instance, CAZy primarily catalogs carbohydrate-active enzymes, with only a subset of auxiliary enzymes (e.g., those acting on lignin) implicated in aromatic compound degradation. Other databases tend to be either incomplete or predominantly focused on nonfungal taxa (e.g., HADEG). Moreover, key biochemical differences between fungal and bacterial central catabolic pathways (e.g., involving 3-carboxymuconolactone and its associated enzymes) are often overlooked (e.g., KEGG and BioCyc), even if being well documented for decades.

To address current gaps and complement existing resources, we present FAMeDB—a curated database of protein entries dedicated to the fungal metabolism of aromatic compounds. Alongside this, we include an R-based bioinformatic workflow designed to facilitate rapid characterization of fungal proteomes regarding their metabolic potential in response to aromatic substrates. This resource serves as a globally accessible platform designed to streamline omics analysis and comparative genomics of fungal aromatics metabolism for the research community.

## Materials and Methods

### Sequence collection

Proteins were mostly identified through literature curation spanning the past 30 years. Primary sources were retrieved via PubMed and Google Scholar using Boolean search strategies over the past 15 years, including terms such as, but not limited to, “fungi“ OR “fungus” combined with specific metabolic intermediates (e.g., “3-hydroxyanthranilate”, “catechol”, “gentisate”, “homogentisate”, “hydroxyquinol”, “methoxyhydroquinone”, and “protocatechuate”). To ensure data fidelity, manual curation involved a multistep validation process: (a) existence of accessible protein sequence; (b) confirmation of functional description with experimental evidence, including biochemical assays, gene expression profiles, functional studies (e.g., using gene deletion mutants), or presence within metabolic gene clusters. Additional orthologs with protein-level evidence in UniProt were obtained via Basic Local Alignment Search Tool (BLAST) searches (E-value thresholds: 10e−10 for sequences <200 amino acids, 10e−50 for others; identity >40%) against the curated SWISS-PROT database, using initially identified entries as queries. These candidates were only integrated into the database following manual curation. Protein sequences and associated descriptive data were retrieved from public repositories, including UniProt [20], National Center for Biotechnology Information [21], and MycoCosm [22]. Complementary annotation of protein entries was performed using eggNOG-mapper v. 2.1.13 (database v. 5.0.2) [23], BlastKOALA and KofamKOALA Release 117.0 [18], and InterProScan5 REST web service [24]. Carbohydrate active enzymes (CAZymes) or auxiliary enzymes as identified in CAZy are not the object of this database and were removed after dbCAN3 search [25], except for the dual tannase / beta-glucosidase BglA, aryl-alcohol oxidase CitC (AA3_2 [auxiliary activity family 3 subfamily 2]), and vanillyl-alcohol oxidase VaoA (AA4 [auxiliary activity family 4]).

### Orthology and database analysis

Proteinortho v. 6.3.6 [26] was used to detect orthologous genes between database entries and different query species proteomes. Default parameters were used except algebraic connectivity was set at 0.3, and identity was set at 40 or at different values as otherwise indicated. Analysis of fungal proteomes with HADEG database was also performed as previously described [17]. For comparison purposes with FAMeDB, the HADEG database was also used directly in orthology analysis. To analyze 150 fungal proteomes, we first selected reference annotated genomes from National Center for Biotechnology Information (<10,000 assembled scaffolds) containing between 5,000 and 30,000 coding genes. From this set, we randomly selected 50 genomes from each of the Ascomycota, Basidiomycota, and non-Dikarya lineages (for accession numbers, see Table S1).

Orthology data were analyzed in R (v. 4.5.1) using the packages tidyverse (v. 2.0.0), scales (v. 1.4.0), svglite (v. 2.2.1), and reshape2 (v. 1.4.4) to identify database-matching hits by pathway and to generate the corresponding tables and plots.

## Results

The FAMeDB curated database currently includes 408 proteins (294 orthology groups; 121 groups with similar domain composition), mostly enzymes but also some transcription factors and transporters (Table S2). The respective pathways range from peripheral catabolism to central metabolism, including some that do not directly deal with aromatic compounds but may be upstream or downstream of them (e.g., quinic acid metabolism) or that belong to secondary metabolism. The pathways include anabolic and catabolic pathways, as it is often impossible to discern molecular components from each other, as they encompass redundancy and plasticity, even more so when considering secondary metabolism.

The database contains entries from 82 species and 56 genera of fungi. Around half are from *Aspergilli* (44%), and the vast majority from Ascomycota (80%). This distribution primarily reflects the fungal species most frequently employed in research, particularly those studied in the context of aromatic compound metabolism.

Among existing resources, the HADEG database offers coverage of aromatic compound metabolism [17]. However, its utility for fungal systems is limited: Only 3 fungal entries are included (although not in aromatics metabolism), and overall orthology and homology between bacterial and fungal enzymes are low. An analysis of 150 random fungal proteomes using the HADEG database yielded only a few specific matches primarily for homogentisate 1,2-dioxygenase HmgA and catechol 1,2-dioxygenase CatA (Fig. S1 and Table S3). The analysis also revealed some nonspecific or unknown hits, including maleylpyruvate isomerase NagL (shows homology to fungal maleylacetoacetate isomerase), 3-oxoadipyl-CoA thiolase PcaF (lacking orthology to characterized Ascomycota counterparts), and 5-carboxymethyl-2-hydroxymuconate semialdehyde dehydrogenase HpcC (homoprotocatechuate degradation, which is not characterized in fungi). The divergence to bacteria is especially pronounced in certain catabolic pathways, such as the fungal-specific 3-carboxymuconolactone route within the protocatechuate branch [7], or the (yet) unique fungal 1,2,3,5-tetrahydroxybenzene central pathway [10]. Attempts to circumvent this limitation by lowering identity thresholds to detect distant homologs often compromise specificity, increasing the likelihood of false positives—proteins that are structurally related but functionally divergent (e.g., superfamily members), a problem that is substantially reduced when using FAMeDB for the analysis of fungal proteomes. Nevertheless, this methodological limitation should be considered when analyzing species outside Ascomycota, given the database’s current bias toward this phylum.

Curation of FAMeDB prioritized entries with robust experimental characterization. Nonetheless, proteins with limited evidence—such as those within conserved gene clusters—were included to encourage further investigation and functional validation. While the primary goal was to facilitate identification of the more divergent catabolic pathways, comprehensive annotation of related metabolic pathways was necessary to ensure contextual accuracy.

Certain enzyme families, such as CAZymes involved in peripheral degradation (e.g., lignin peroxidases), are better accessed through specialized resources like CAZy and dbCAN3 [25,27]. Contrarily, conserved biosynthetic pathways (e.g., coenzyme Q10 synthesis) were retained in the database, as their constituent genes may have been repurposed for novel functions. These distant paralogs are particularly prone to misannotation, especially when comparing them across distantly related taxa.

Another conserved pathway—the nicotinate metabolism—was also included due to its potential intersection with aromatic catabolism via the upper 3-hydroxyanthranilate pathway, which may compensate for the absence of the lower pathway in certain species such as those of genus *Candida* [9,28]. Although further research is needed, its relevance warrants inclusion. On the other hand, Basidiomycota generally retain NAD biosynthesis and salvage genes but lack many of the nicotinate degradation genes [29].

Pathway annotation also required careful consideration of phylum-specific differences. For instance, Basidiomycota variants (different gene architecture or orthology) of the catechol and protocatechuate branch in the 3-oxoadipate pathway are supported primarily by conserved gene cluster evidence, including protocatechuate dioxygenase [8]. These differences, though preliminary, should not be overlooked. Despite the genetic differences, they were annotated in the corresponding pathway as there is no evidence of biochemical distinctions between fungal phyla nor is there evidence that both gene compositions could be present in a single organism.

The gentisate-like pathway presents additional challenges. It lacks a maleylpyruvate isomerase, and its functional role remains unclear despite genomic co-occurrence with the canonical gentisate pathway [8,30]. Transcriptomic data indicates upregulation in gentisate- or lignin-containing media [31], but definitive functional validation is pending. Notably, Basidiomycota gentisate-like clusters also omit this isomerase, suggesting that it may be nonessential or involved in detoxification of metabolites of gentisate derivatives. Distinct catalytic properties of gentisate 1,2-dioxygenases from these clusters [32] support the hypothesis of isoenzyme specialization. Nevertheless, the distinction was only made in the annotation of the peripheral pathways, as the central pathway proteins are conserved and cannot be distinguished by the current methodology.

Functional or enzymatic redundancy is another important consideration. For example, maleylacetate reductase plays roles in both hydroxyquinol and methoxyhydroquinone pathways [12,33]. Gallate 1-monooxygenase (decarboxylating) can play a role upstream of the 1,2,3,5-tetrahydroxybenzene pathway or the hydroxyquinol branch, as it also can metabolize, e.g., protocatechuate [10,12,34]. The 3-carboxy-*cis*,*cis*-muconate cyclase paralog in Basidiomycota has a putative role as a *cis*,*cis*-muconate cycloisomerase even if joined together with 3-oxoadipate-enol lactonase in a multifunctional enzyme [8]. Similarly, enzymes in CoA-dependent pathways, such as those in the 3-oxoadipate pathway or β-oxidative routes for hydroxycinnamic acids, are prone to produce false positives or misannotation using FAMeDB analysis due to high family-level redundancy. For instance, the high sequence homology between the orthologs 3-oxoadipyl-CoA thiolase KctA (3-oxoadipate pathway) and 3-ketoacyl CoA thiolase KatA (cinnamic acids CoA-dependent beta-oxidative pathway) often leads to misannotation, as the methodology described here lacks the necessary resolution to fully distinguish them, even within the same phyla.

**Fig. 2.**
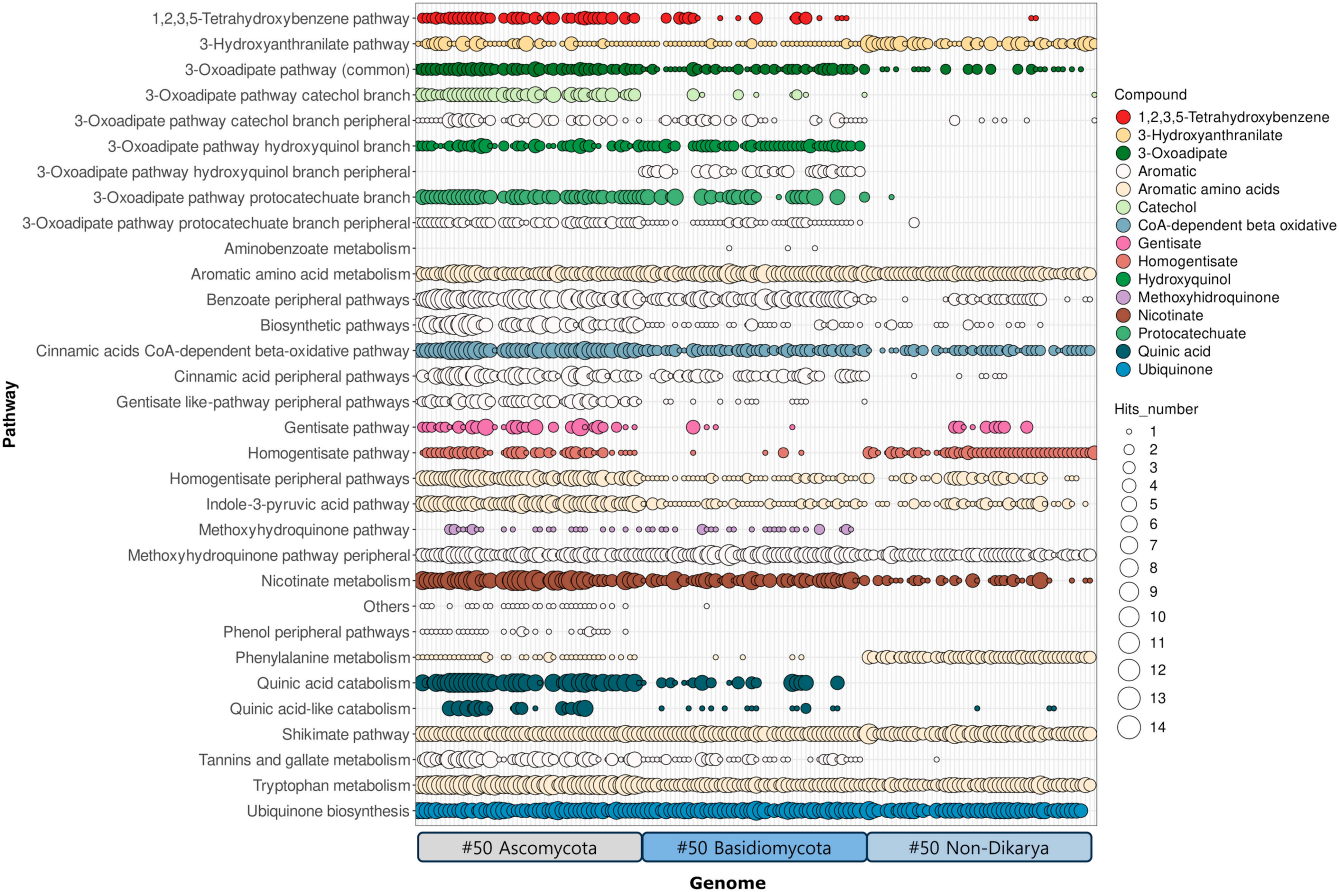
FAMeDB analysis of 150 random fungal proteomes. The number of database hits annotated per pathway is displayed using a bubble plot. Bubbles are colored according to compound. The rightmost column provides a direct comparison of matches obtained against the Hydrocarbon Aerobic Degradation Enzymes and Genes (HADEG) database. Genomes are sorted alphabetically by species name in their corresponding taxonomic group (see Table S1). Results shown consider a minimum sequence identity of 40% in the orthology analysis step.

These nuances in database construction should inform interpretation of results derived from FAMeDB. Nevertheless, the application of the database and its associated tools to 150 randomly selected fungal genomes across multiple phyla (Fig. 2 and Tables S4 to S6) demonstrates its capacity to deliver rapid, informative insights into fungal aromatic metabolism.

**Fig. 3.**
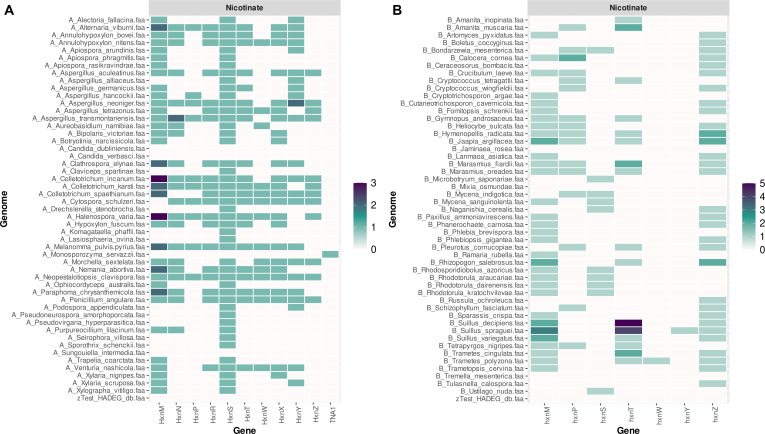
FAMeDB analysis of nicotinate metabolism in 50 random proteomes of Ascomycota (A) and 50 of Basidiomycota (B). Genes of nicotinate metabolism and in particular nicotinate hydroxylase HnxS are less frequently present in Basidiomycota in comparison to Ascomycota genomes. The number of database hits of the nicotinate metabolism annotated per proteome is displayed using a heatmap plot. The last row provides a direct comparison of matches obtained against the Hydrocarbon Aerobic Degradation Enzymes and Genes (HADEG) database. Results shown consider a minimum sequence identity of 40% in the orthology analysis step.

**Fig. 4.**
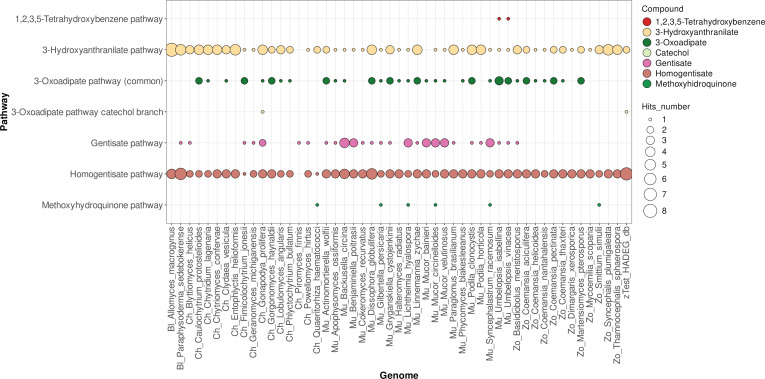
FAMeDB analysis of the central pathways for aromatic compounds metabolism of 50 random fungal proteomes of non-Dykaria. The number of database hits annotated per pathway is displayed using a bubble plot. Bubbles are colored according to compound. The rightmost column provides a direct comparison of matches obtained against the Hydrocarbon Aerobic Degradation Enzymes and Genes (HADEG) database. Results shown consider a minimum sequence identity of 40% in the orthology analysis step.

Overall, the analysis of the results obtained with 150 fungal proteomes (Fig. 2, Figs. S2 to S6, and Tables S4 to S6) quickly indicates several key differences between the 3 taxonomic groups under analysis. Ascomycota genomes generally have a larger repertoire of central pathways, although caution should be exercised as more research has been conducted on this fungal taxon. Consequently, some proteins may not be properly identified for the other taxa in the current version of FAMeDB, as the workflow described here relies on homology-based identification using currently characterized proteins. It can also be observed that, in general, some central pathways are less frequently present in Basidiomycota species and that they have limited nicotinate metabolism. The last is better observed in a heatmap plot of the presence of the genes of this pathway (Fig. 3), which shows that the HnxS nicotinate hydroxylase is less frequently present, as are the other genes, in Basidiomycota than in Ascomycota. Non-Dikarya fungi have a more limited set of catabolic central pathways—at least among those currently known (Figs. 2 and 4 and Figs. S2 to S6). Nonetheless, non-Dikarya fungi frequently possess the 3-hydroxyanthranilate and homogentisate pathways, which may be important for the metabolism of aromatic amino acids.

Detailed results are available via the tables automatically generated during FAMeDB analysis (see database webpage for an example with all files). These outputs simplify the extraction of protein IDs based on query species and pathways (Table S6), allowing for seamless export for downstream applications, such as RNA sequencing enrichment analysis.

Setting the identity at 40% in the orthology step allows a broader identification of hits while keeping false positives relatively low, while permitting to withstand the wide differences between species in the fungi kingdom. The identification of a single hit or multiple hits for the same protein is common and reflects the expected redundancy. Therefore, the presence of a certain pathway should only be admitted if 2 or more different enzymes are identified. For non-Dikarya fungi, a 30% identity threshold can help capture more divergent orthologues, though these candidates require additional validation. Conversely, for Ascomycota datasets, a 50% identity cutoff may increase specificity and be more appropriate depending on the characteristics of the taxa under analysis.

## Discussion

FAMeDB was shown here to be a useful resource for analyzing proteomes of all fungal lineages in relation to their potential for aromatic metabolism. The database currently has a bias toward Ascomycota, but the results indicate that this does not fundamentally compromise analyses across taxonomically diverse fungi. This is evidenced by the identification of all 8 central pathways within the less studied Basidiomycota or non-Dikarya lineages, alongside the near-universal detection of highly conserved pathways like shikimate and ubiquinone biosynthesis. It is important to mention that all the entries for these conserved pathways are from Ascomycota. However, since the methodology described here relies on known orthology, we cannot rule out functional convergence or the existence of novel metabolic strategies—particularly in poorly studied non-Dikarya fungi or for anaerobic environments [1,3]. Continuous updates to the database to reflect advances on less studied lineages will ensure greater robustness over time.

For Dikarya, our results are consistent with a previous study based on the detection of gene clusters [8]. It reported that Ascomycota generally harbor a broader diversity of central pathways, whereas Basidiomycota tend to rely on fewer pathways, likely channeling metabolites into a smaller set—a strategy previously proposed for the hydroxyquinol branch of the 3-oxoadipate pathway [33]. In non-Dikarya, the absence of several pathways aligns with the characteristically low abundance of intradiol ring-cleavage dioxygenases (IPR015889; catechol, hydroxyquinol, and protocatechuate branches) or open-ring DUF3550 (IPR021889; 1,2,3,5-tetrahydroxybenzene pathway) in their proteomes (Figs. S7 and S8).

The more recently characterized central pathways for methoxyhydroquinone and 1,2,3,5-tetrahydroxybenzene [10–12] appear to be frequently present across Dikarya (Fig. 2). The latter pathway, in particular, seems to be almost universally conserved in Ascomycota, with exceptions largely limited to species lacking other central pathways (e.g., some members of the *Candida* genus) or possessing only a very reduced set of pathways (e.g., *Alectoria fallacina* and *Xylographa vitiligo*). As methoxyhydroquinone and 1,2,3,5-tetrahydroxybenzene pathways are expected to play key roles in lignin utilization—supporting the degradation of hydroxyphenyl/guaiacyl and syringyl units, respectively—their distribution raises an important question. If these pathways are comparatively rare in Basidiomycota (i.e., in the random dataset used), it remains unclear whether this phylum relies more heavily on the hydroxyquinol and protocatechuate branches of the 3-oxoadipate pathway (Fig. 2) or whether distinct molecular or metabolic strategies exist that have yet to be characterized.

Non-Dikarya fungi generally encode fewer central pathways overall (Figs. 2 and 4). Even so, the 3-hydroxyanthranilate and homogentisate pathways—both likely involved in the catabolism of aromatic amino acids—are broadly distributed in these lineages, unlike what is observed in Basidiomycota. This uneven distribution also helps explain why non-Dikarya taxa have historically received less attention in studies of aromatic compound metabolism.

About one-third of the FAMeDB entries (143; 35%) lack annotation by KEGG tools, such as KofamKOALA or BlastKOALA (Table S2). Among the 8 central pathways, fewer than half of the FAMeDB entries are correctly annotated (50 of 105). This limitation affects not only recently characterized pathways [10–12] for methoxyhydroquinone and 1,2,3,5-tetrahydroxybenzene—which show no accurate predictions—but also long-established ones. For example, in the catechol branch, only 1 of the 4 core enzymes (catechol 1,2-dioxygenase) is correctly identified. In the gentisate pathway, only the gentisate 1,2-dioxygenase is recovered among its 3 core enzymes. For the hydroxyquinol pathway, the analysis performs slightly better, with both enzymes correctly identified, but only by KofamKOALA; even then, only half of the proteins for maleylacetate reductase and 80% for hydroxyquinol 1,2-dioxygenase are detected. Overall, these results highlight how general annotation resources can fall short when applied to specific metabolic analyses.

FAMeDB is inherently limited by the current understanding of fungal aromatic catabolism, including the input from its curators. To remain relevant and helpful, it should be constantly updated, preferably with the help of the research community.

The analysis methodology reported in this work is highly dependent on how well annotated the genomes under study are or even if annotated at all at the gene level. Absent or wrongly annotated genes (e.g., shorter) in a genome annotation can give the suggestion that a specific pathway may not be present. In a future development, this can be mitigated if the analysis can be performed on the genome sequence directly. Alternatively, integrating recent or emerging tools that offer faster and more accurate fungal genome annotation would substantially improve data quality prior to analysis with FAMeDB.

In conclusion, FAMeDB enables the rapid and informative analysis of fungal genomes, shedding light on their aromatic metabolism. This platform is suited both for species-level analyses, including genome and transcriptome interrogation, and for comparative studies across multiple species to investigate evolutionary patterns. Consequently, this toolset (schematically represented in Fig. S9) can be used to integrate knowledge and accelerate future research across various fields that study aromatic metabolism.

## Data Availability

All data associated with FAMeDB—including protein sequences and annotations, pathway descriptive table, and accompanying R script—is publicly available in the GitHub repository (https://github.com/SilvaPereiraLab/FAMeDB).

## References

[1] Boll M, Fuchs G, Heider J. Anaerobic oxidation of aromatic compounds and hydrocarbons. Curr Opin Chem Biol. 2002;6(5):604–611. 12413544 10.1016/s1367-5931(02)00375-7

[2] Lubbers RJM. An updated perspective on the aromatic metabolic pathways of plant-derived homocyclic aromatic compounds in *Aspergillus niger*. Microorganisms. 2025;13(8):1718. 40871222 10.3390/microorganisms13081718 PMC12388069

[3] Kovács E, Szűcs C, Juhász-Erdélyi A, Bagi Z, Kovács KL. Anaerobic fungi: Effective warriors in lignocellulosic biomass degradation and fermentation. FEMS Microbiol Ecol. 2025;101(11):fiaf108. 41129402 10.1093/femsec/fiaf108 PMC12586995

[4] Mäkelä MR, Marinović M, Nousiainen P, Liwanag AJM, Benoit I, Sipilä J, Hatakka A, De Vries RP, Hildén KS. Aromatic metabolism of filamentous fungi in relation to the presence of aromatic compounds in plant biomass. Adv Appl Microbiol. 2015;91:63–137. 25911233 10.1016/bs.aambs.2014.12.001

[5] Martins I, Hartmann DO, Alves PC, Martins C, Garcia H, Leclercq CC, Ferreira R, He J, Renaut J, Becker JD, et al. Elucidating how the saprophytic fungus *Aspergillus nidulans* uses the plant polyester suberin as carbon source. BMC Genomics. 2014;15:613. 25043916 10.1186/1471-2164-15-613 PMC4117967

[6] Hammerbacher A, Schmidt A, Wadke N, Wright LP, Schneider B, Bohlmann J, Brand WA, Fenning TM, Gershenzon J, Paetz C. A common fungal associate of the spruce bark beetle metabolizes the stilbene defenses of Norway spruce. Plant Physiol. 2013;162(3):1324–1336. 23729780 10.1104/pp.113.218610 PMC3707561

[7] Dilokpimol A, Visser J, Mäkelä MR, Hildén KS, De Vries RP. A comparison between the homocyclic aromatic metabolic pathways from plant-derived compounds by bacteria and fungi. Biotechnol Adv. 2019;37(7): Article 107396. 31075306 10.1016/j.biotechadv.2019.05.002

[8] Martins TM, Martins C, Silva Pereira C. Multiple degrees of separation in the central pathways of the catabolism of aromatic compounds in fungi belonging to the Dikarya sub-Kingdom. Adv Microb Physiol. 2019;75:177–203. 31655737 10.1016/bs.ampbs.2019.07.003

[9] Martins TM, Martins C, Guedes P, Silva Pereira C. Twists and turns in the salicylate catabolism of *Aspergillus terreus*, revealing new roles of the 3-hydroxyanthranilate pathway. mSystems. 2021;6(1):e00230–e00220. 33500329 10.1128/mSystems.00230-20 PMC7842363

[10] Martins TM, Bento A, Martins C, Tomé AS, Moreira CJS, Silva Pereira C. Bringing up to date the toolkit for the catabolism of aromatic compounds in fungi: The unexpected 1,2,3,5-tetrahydroxybenzene central pathway. Microb Biotechnol. 2024;17(1): Article e14371. 38064205 10.1111/1751-7915.14371 PMC10832562

[11] Kato H, Takahashi Y, Suzuki H, Ohashi K, Kawashima R, Nakamura K, Sakai K, Hori C, Takasuka TE, Kato M, et al. Identification and characterization of methoxy- and dimethoxyhydroquinone 1,2-dioxygenase from *Phanerochaete chrysosporium*. Appl Environ Microbiol. 2024;90(2):e01753–e01723. 38259078 10.1128/aem.01753-23 PMC10880611

[12] Dilokpimol A, Nousiainen PA, Cioc RC, Visser J, Bruijnincx PCA, De Vries RP. Vanillic acid and methoxyhydroquinone production from guaiacyl units and related aromatic compounds using *Aspergillus niger* cell factories. Microb Cell Fact. 2021;20(1):151. 34344380 10.1186/s12934-021-01643-x PMC8336404

[13] Shettigar M, Balotra S, Kasprzak A, Pearce SL, Lacey MJ, Taylor MC, Liu J-W, Cahill D, Oakeshott JG, Pandey G. Oxidative catabolism of (+)-pinoresinol is initiated by an unusual flavocytochrome encoded by translationally coupled genes within a cluster of (+)-pinoresinol-coinduced genes in *Pseudomonas* sp. strain SG-MS2. Appl Environ Microbiol. 2020;86(10):e00375–e00320. 32198167 10.1128/AEM.00375-20 PMC7205488

[14] Silva COG, Bapat NA, Bourmaud CL, Jensen CN, de Bueren JB, Luterbacher J, Meyer AS, van Erven G, van Berkel WJH, Kabel MA, et al. Oxygenation and oxidation of lignin model dimers by fungal *ortho*-methoxyphenolases. J Am Chem Soc. 2026;148(6):6727–6738. 41640198 10.1021/jacs.5c22901 PMC12921834

[15] Lubbers RJM, Dilokpimol A, Visser J, De Vries RP. *Aspergillus niger* uses the peroxisomal CoA-dependent β-oxidative genes to degrade the hydroxycinnamic acids caffeic acid, ferulic acid, and *p*-coumaric acid. Appl Microbiol Biotechnol. 2021;105(10):4199–4211. 33950281 10.1007/s00253-021-11311-0 PMC8140964

[16] Drula E, Garron M-L, Dogan S, Lombard V, Henrissat B, Terrapon N. The carbohydrate-active enzyme database: Functions and literature. Nucleic Acids Res. 2022;50(D1):D571–D577. 34850161 10.1093/nar/gkab1045 PMC8728194

[17] Rojas-Vargas J, Castelán-Sánchez HG, Pardo-López L. HADEG: A curated hydrocarbon aerobic degradation enzymes and genes database. Comput Biol Chem. 2023;107: Article 107966. 37778093 10.1016/j.compbiolchem.2023.107966

[18] Kanehisa M, Furumichi M, Sato Y, Matsuura Y, Ishiguro-Watanabe M. KEGG: Biological systems database as a model of the real world. Nucleic Acids Res. 2025;53(D1):D672–D677. 39417505 10.1093/nar/gkae909 PMC11701520

[19] Karp PD, Billington R, Caspi R, Fulcher CA, Latendresse M, Kothari A, Keseler IM, Krummenacker M, Midford PE, Ong Q, et al. The BioCyc collection of microbial genomes and metabolic pathways. Brief Bioinform. 2019;20(4):1085–1093. 29447345 10.1093/bib/bbx085 PMC6781571

[20] The UniProt Consortium. UniProt: The universal protein knowledgebase in 2025. Nucleic Acids Res. 2025;51(D1):D609–D617. 10.1093/nar/gkae1010 PMC11701636 39552041

[21] Goldfarb T, Kodali VK, Pujar S, Brover V, Robbertse B, Farrell CM, Oh D-H, Astashyn A, Ermolaeva O, Haddad D, et al. NCBI RefSeq: Reference sequence standards through 25 years of curation and annotation. Nucleic Acids Res. 2025;53(D1):D243–D257. 39526381 10.1093/nar/gkae1038 PMC11701664

[22] Grigoriev IV, Nikitin R, Haridas S, Kuo A, Ohm R, Otillar R, Riley R, Salamov A, Zhao X, Korzeniewski F, et al. MycoCosm portal: Gearing up for 1000 fungal genomes. Nucleic Acids Res. 2014;42:D699–D704. 24297253 10.1093/nar/gkt1183 PMC3965089

[23] Hernández-Plaza A, Szklarczyk D, Botas J, Cantalapiedra CP, Giner-Lamia J, Mende DR, Kirsch R, Rattei T, Letunic I, Jensen LJ, et al. eggNOG 6.0: Enabling comparative genomics across 12 535 organisms. Nucleic Acids Res. 2023;51(D1):D389–D394. 36399505 10.1093/nar/gkac1022 PMC9825578

[24] Blum M, Andreeva A, Florentino LC, Chuguransky SR, Grego T, Hobbs E, Pinto BL, Orr A, Paysan-Lafosse T, Ponamareva I, et al. InterPro: The protein sequence classification resource in 2025. Nucleic Acids Res. 2025;53(D1):D444–D456. 39565202 10.1093/nar/gkae1082 PMC11701551

[25] Zheng J, Ge Q, Yan Y, Zhang X, Huang L, Yin Y. Db CAN3: Automated carbohydrate-active enzyme and substrate annotation. Nucleic Acids Res. 2023;51(W1):W115–W121. 37125649 10.1093/nar/gkad328 PMC10320055

[26] Klemm P, Stadler PF, Lechner M. Proteinortho6: Pseudo-reciprocal best alignment heuristic for graph-based detection of (co-)orthologs. Front Bioinform. 2023;3:1322477. 38152702 10.3389/fbinf.2023.1322477 PMC10751348

[27] Lombard V, Golaconda Ramulu H, Drula E, Coutinho PM, Henrissat B. The carbohydrate-active enzymes database (CAZy) in 2013. Nucleic Acids Res. 2014;42:D490–D495. 24270786 10.1093/nar/gkt1178 PMC3965031

[28] Han T-L, Tumanov S, Cannon RD, Villas-Boas SG. Metabolic response of *Candida albicans* to phenylethyl alcohol under hyphae-inducing conditions. PLOS One. 2013;8(8): Article e71364. 23951145 10.1371/journal.pone.0071364 PMC3741116

[29] Bokor E, Flipphi M, Kocsubé S, Ámon J, Vágvölgyi C, Scazzocchio C, Hamari Z. Genome organization and evolution of a eukaryotic nicotinate co-inducible pathway. Open Biol. 2021;11(9): Article 210099. 34582709 10.1098/rsob.210099 PMC8478523

[30] Greene GH, McGary KL, Rokas A, Slot JC. Ecology drives the distribution of specialized tyrosine metabolism modules in fungi. Genome Biol Evol. 2014;6(1):121–132. 24391152 10.1093/gbe/evt208 PMC3914699

[31] Poirier W, Ravenel K, Bouchara J-P, Giraud S. Lower funneling pathways in *Scedosporium* species. Front Microbiol. 2021;12: Article 630753. 34276578 10.3389/fmicb.2021.630753 PMC8283699

[32] Semana P, Powlowski J. A chimeric variant of two gentisate 1,2-dioxygenases with improved catalytic activity, altered structural conformation and substrate specificity. FASEB J. 2021;35(S1): 10.1096/fasebj.2021.35.S1.00262

[33] Kuatsjah E, Schwartz A, Zahn M, Tornesakis K, Kellermyer ZA, Ingraham MA, Woodworth SP, Ramirez KJ, Cox PA, Pickford AR, et al. Biochemical and structural characterization of enzymes in the 4-hydroxybenzoate catabolic pathway of lignin-degrading white-rot fungi. Cell Rep. 2024;43(12): Article 115002. 39589922 10.1016/j.celrep.2024.115002

[34] Lubbers RJM, De Vries RP. Production of protocatechuic acid from *p*-hydroxyphenyl (H) units and related aromatic compounds using an *Aspergillus niger* cell factory. MBio. 2021;12(3):e00391–e00321. 34154420 10.1128/mBio.00391-21 PMC8262893

