# Supplementary material for: FAMeDB: A Curated Database for the Analysis of Fungal Aromatic Compound Metabolism
Source: Comput Struct Biotechnol J. 2026 Apr 20;35(1):0049. doi: 10.34133/csbj.0049 (PMC13094099; doi:10.34133/csbj.0049)
Supplement: Supplementary 1 — Figs S1 to S9 Tables S1 to S6 [file csbj.0049.f1.zip › FAMeDB_supplementary_figures_R1.pdf]

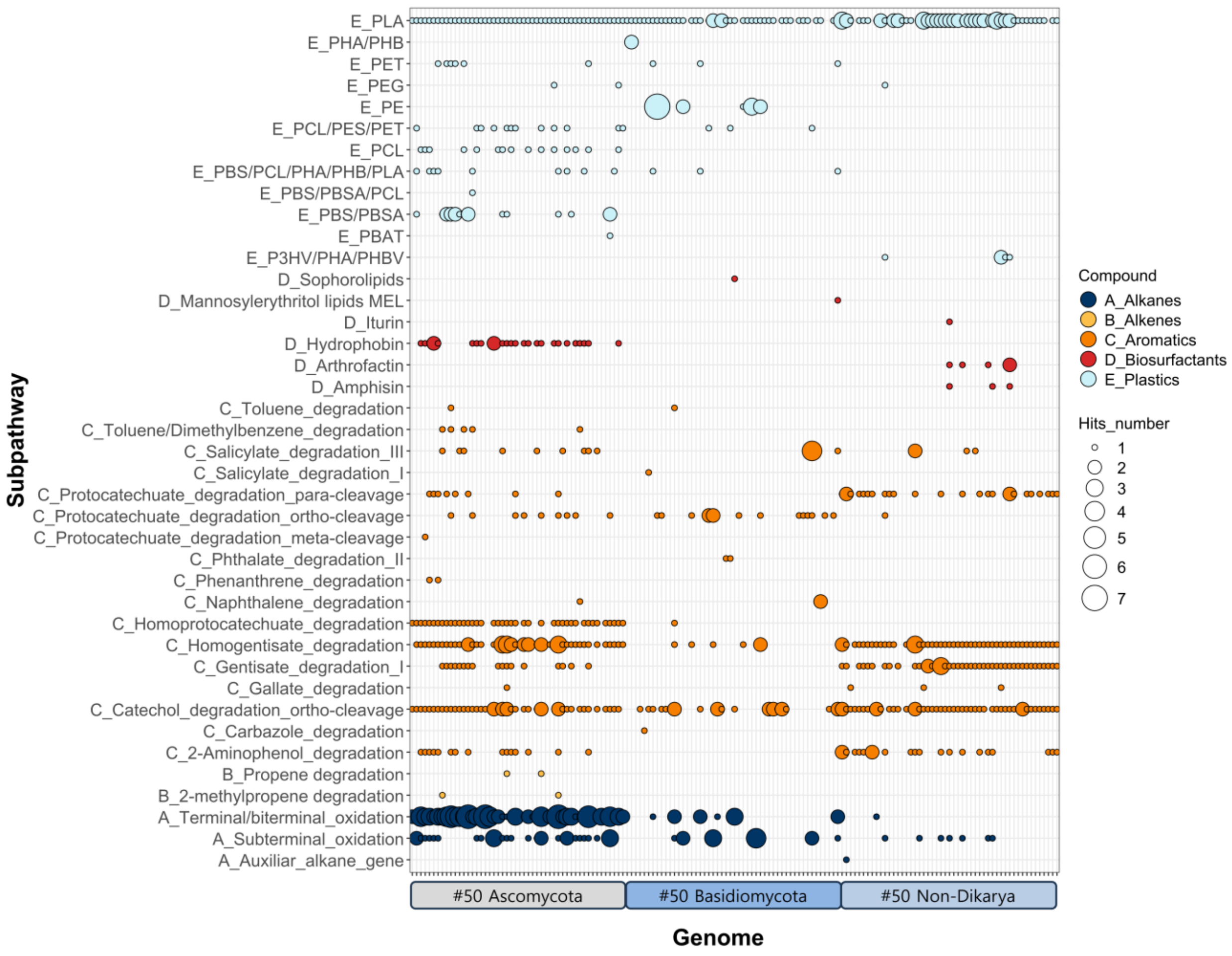

Figure S1. HADEG database analysis of 150 random fungal genomes. The number of database hits annotated per pathway is displayed using a bubble plot. The rightmost column provides a direct comparison of matches obtained against FAMeDB. Genomes are sorted alphabetically by species name in their corresponding taxonomic group (see Table S1). Results shown consider a minimum sequence identity of 40% in the orthology analysis step.

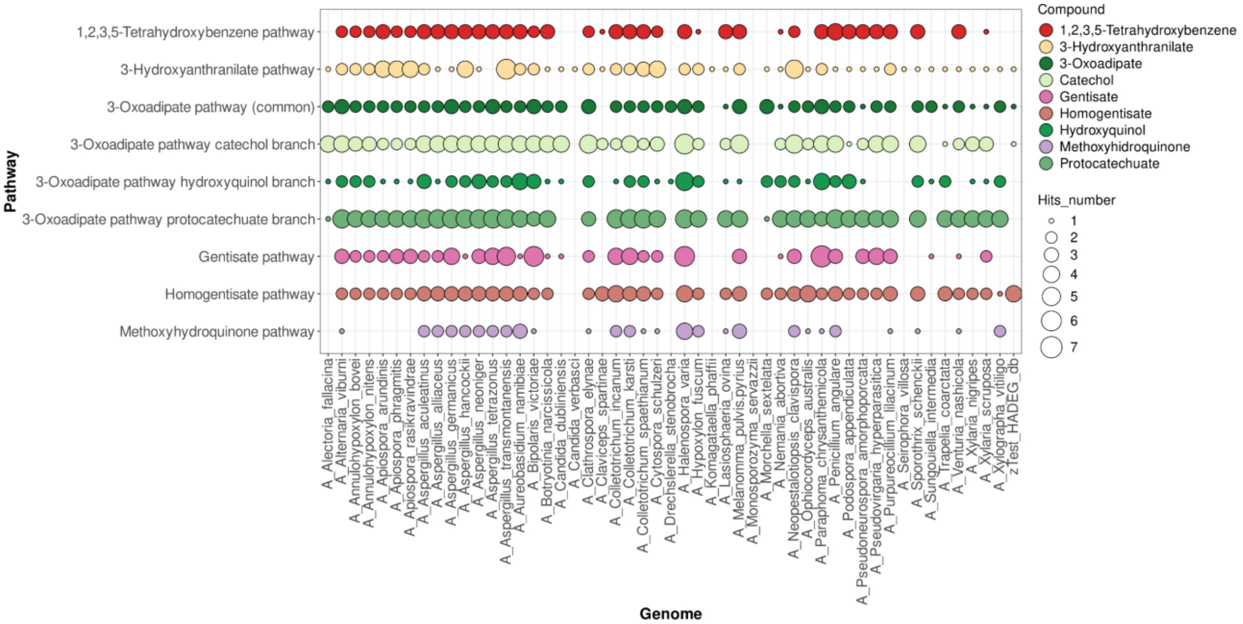

Figure S2. FAMeDB bubble plot analysis of the central pathways for aromatic compounds catabolism of fifty random fungal proteomes of Ascomycota. The number of database hits annotated per pathway is displayed using a bubble plot. Bubbles are colored according to compound. The rightmost column provides a direct comparison of matches obtained against HADEG database. Results shown consider a minimum sequence identity of 40% in the orthology analysis step.

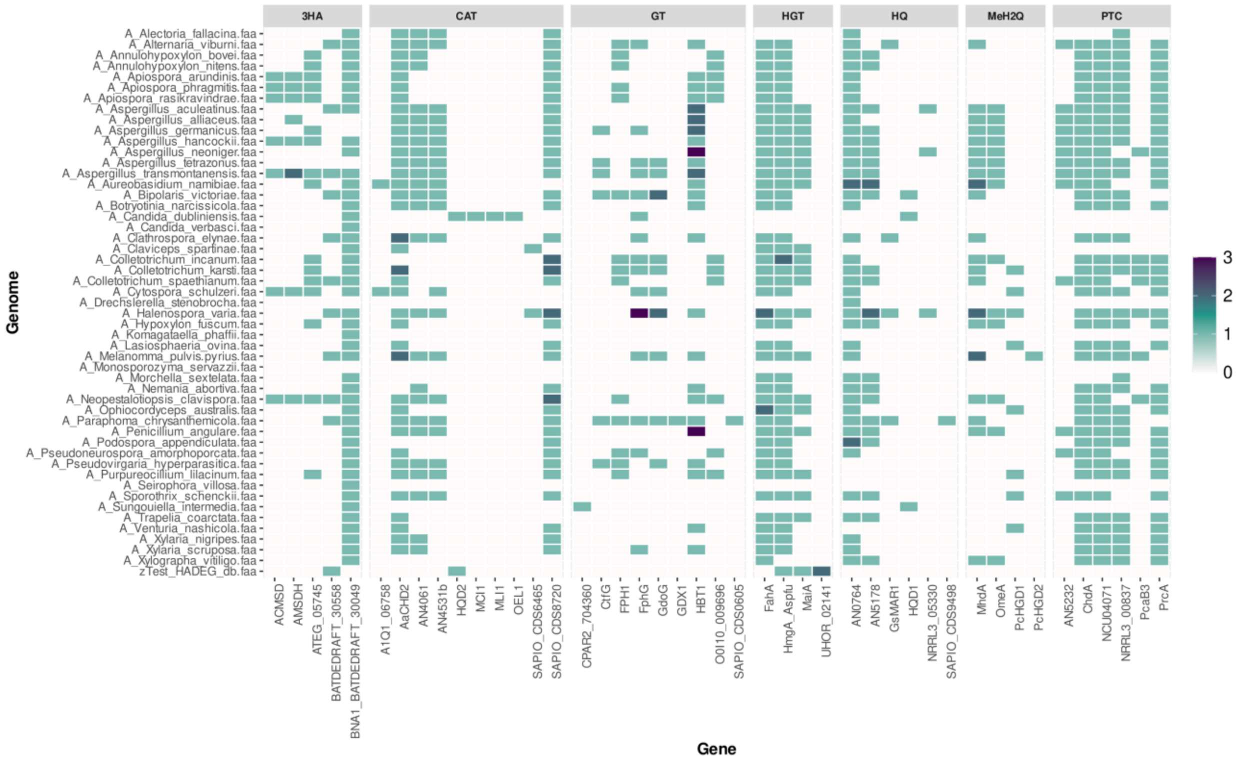

Figure S3. FAMeDB heatmap analysis of central pathways for aromatic compounds catabolism in fifty random proteomes of Ascomycota. The number of database hits of the central pathways annotated per proteome and gene (representing an ortholog group) is displayed using a heatmap plot. The last row provides a direct comparison of matches obtained against HADEG database. Results shown consider a minimum sequence identity of 40% in the orthology analysis step. Abbreviations: 1235TeHB - 1,2,3,5-tetrahydroxybenzene, 3HAO - 3-hydroxyanthranilate, CAT - catechol, GT - gentisate, HGT - homogentisate, HQ - hydroxyquinol, MeH2Q - methoxyhydroquinone, and PTC – protocatechuate.

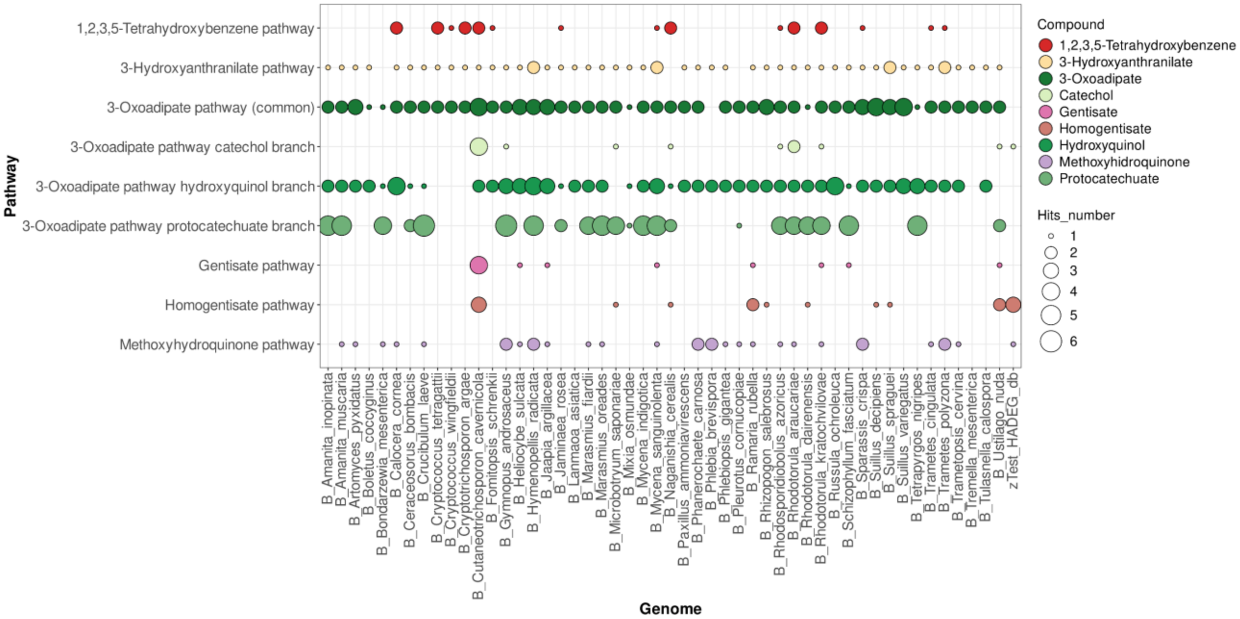

Figure S4. FAMeDB bubble plot analysis of the central pathways for aromatic compounds catabolism of fifty random fungal proteomes of Basidiomycota. The number of database hits annotated per pathway is displayed using a bubble plot. Bubbles are colored according to compound. The rightmost column provides a direct comparison of matches obtained against HADEG database. Results shown consider a minimum sequence identity of 40% in the orthology analysis step.

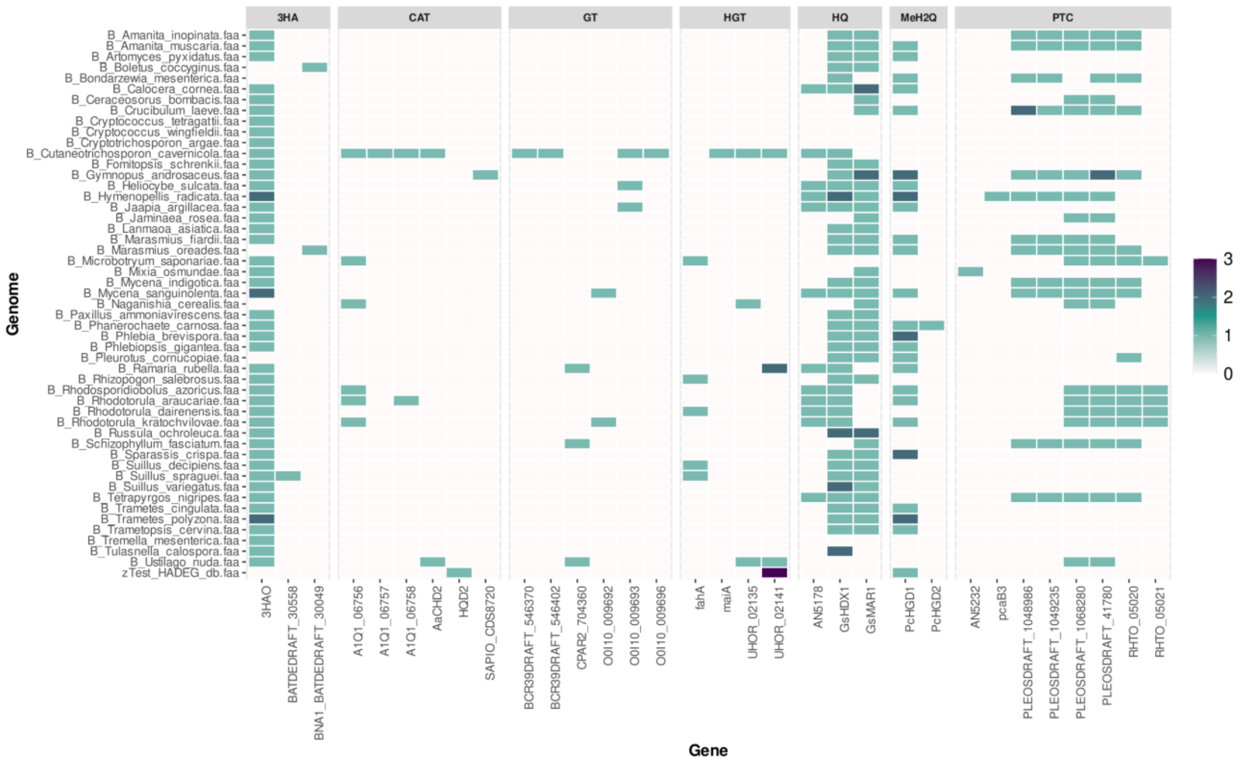

Figure S5. FAMeDB heatmap analysis of central pathways in fifty random proteomes of Basidiomycota. The number of database hits of the central pathways annotated per proteome and gene (representing an ortholog group) is displayed using a heatmap plot. The last row provides a direct comparison of matches obtained against HADEG database. Results shown consider a minimum sequence identity of 40% in the orthology analysis step. Abbreviations: 1235TeHB - 1,2,3,5-tetrahydroxybenzene, 3HAO - 3-hydroxyanthranilate, CAT - catechol, GT - gentisate, HGT - homogentisate, HQ - hydroxyquinol, MeH2Q - methoxyhydroquinone, and PTC – protocatechuate.

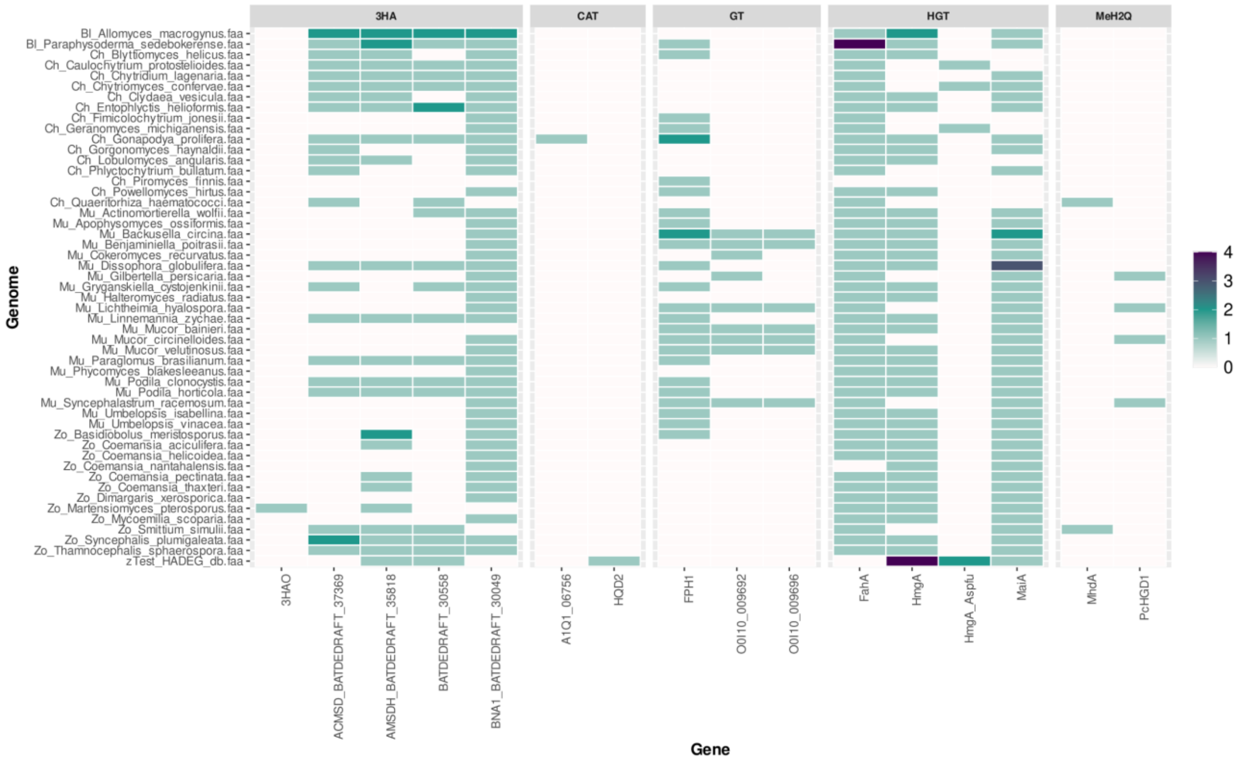

Figure S6. FAMeDB heatmap analysis of central pathways in fifty random proteomes of non-Dikarya. The number of database hits of the central pathways annotated per proteome and gene (representing an ortholog group) is displayed using a heatmap plot. The last row provides a direct comparison of matches obtained against HADEG database. Results shown consider a minimum sequence identity of 40% in the orthology analysis step. Abbreviations: 1235TeHB - 1,2,3,5-tetrahydroxybenzene, 3HAO - 3-hydroxyanthranilate, CAT - catechol, GT - gentisate, HGT - homogentisate, HQ - hydroxyquinol, MeH2Q - methoxyhydroquinone, and PTC – protocatechuate.

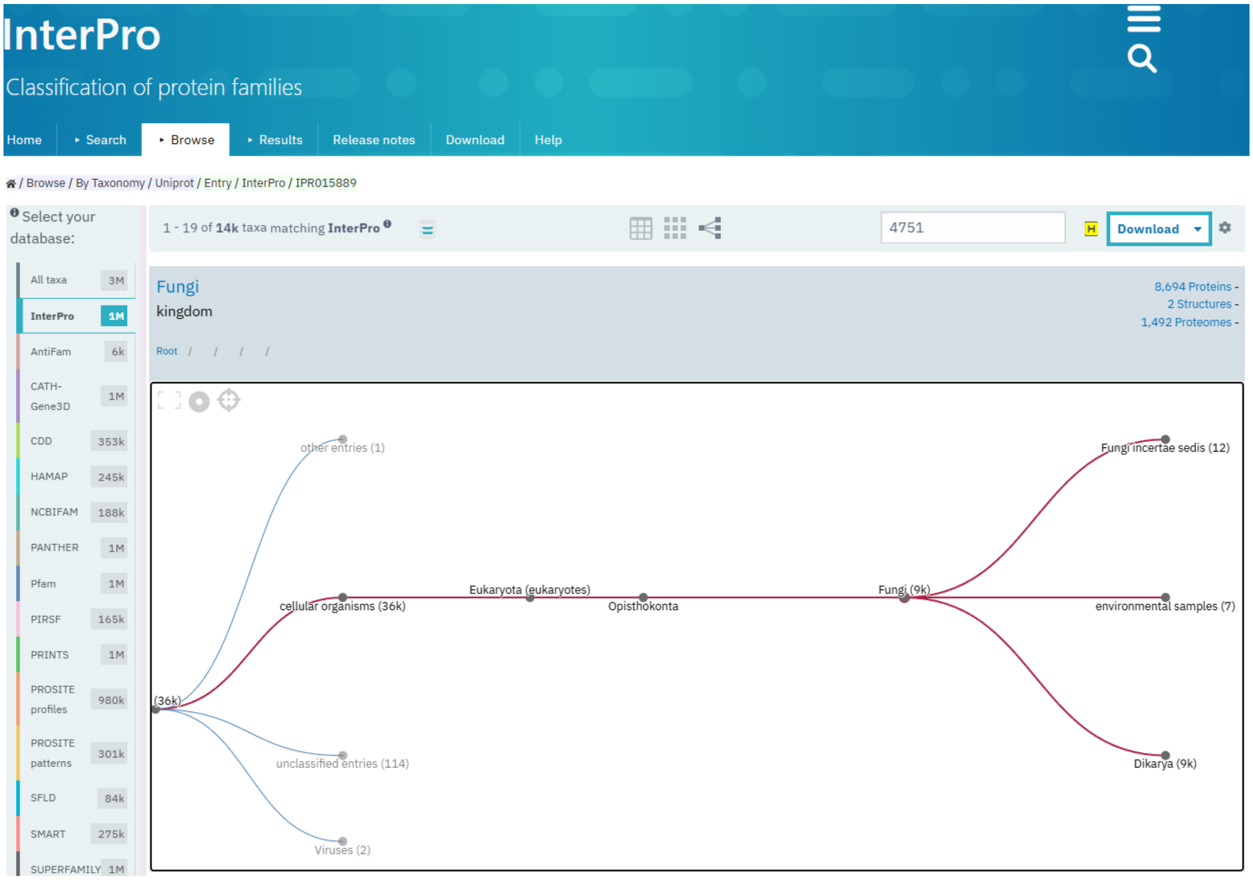

Figure S7. Taxonomic distribution of the InterPro domain IPR015889 Intradiol ring-cleavage dioxygenase, core in Fungi. Twelve hits for "Fungi incertae sedis" or non-Dikarya (173 proteomes) in comparison to 8675 for Dikarya (1319 proteomes). Data obtained at InterPro website (https://www.ebi.ac.uk/interpro/).

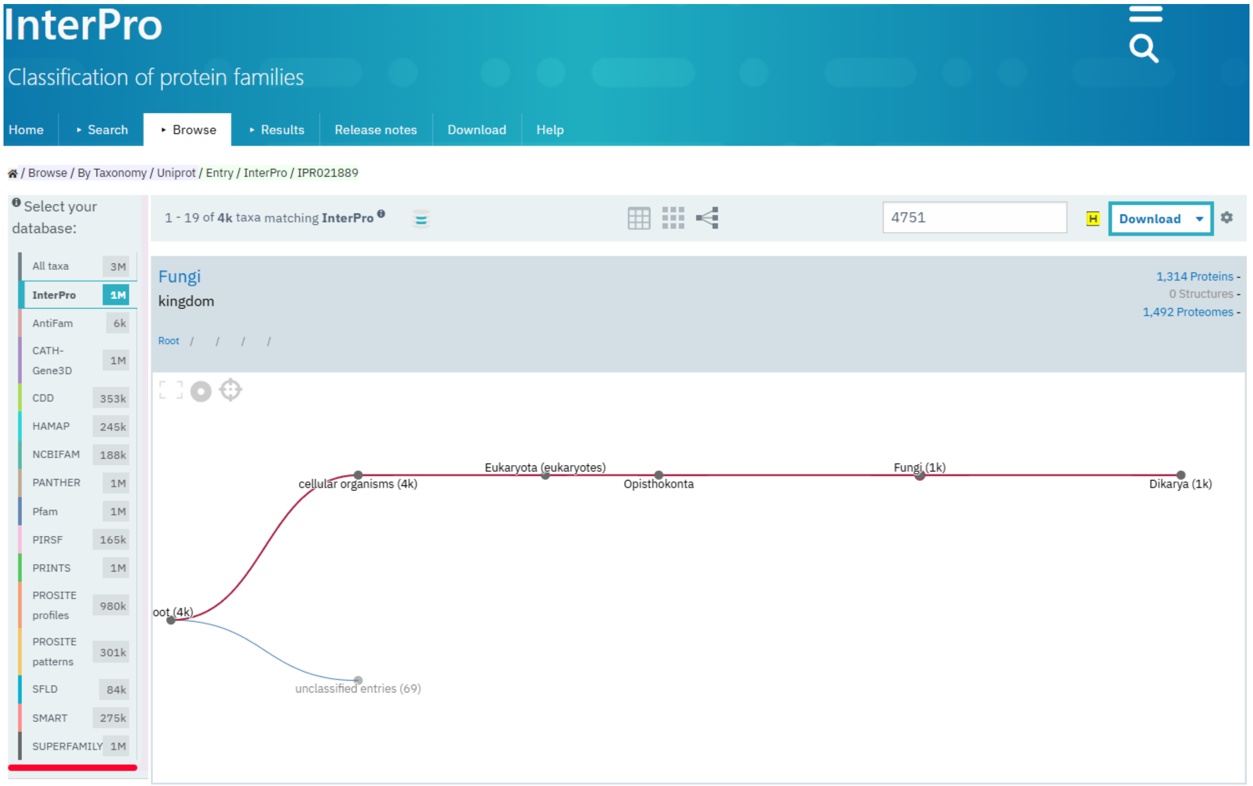

Figure S8. Taxonomic distribution of the InterPro domain IPR021889 Protein of unknown function DUF3500 in Fungi. No hits for "Fungi incertae sedis" or non-Dikarya in comparison to 1314 for Dikarya (1319 proteomes). Data obtained at InterPro website (https://www.ebi.ac.uk/interpro/).

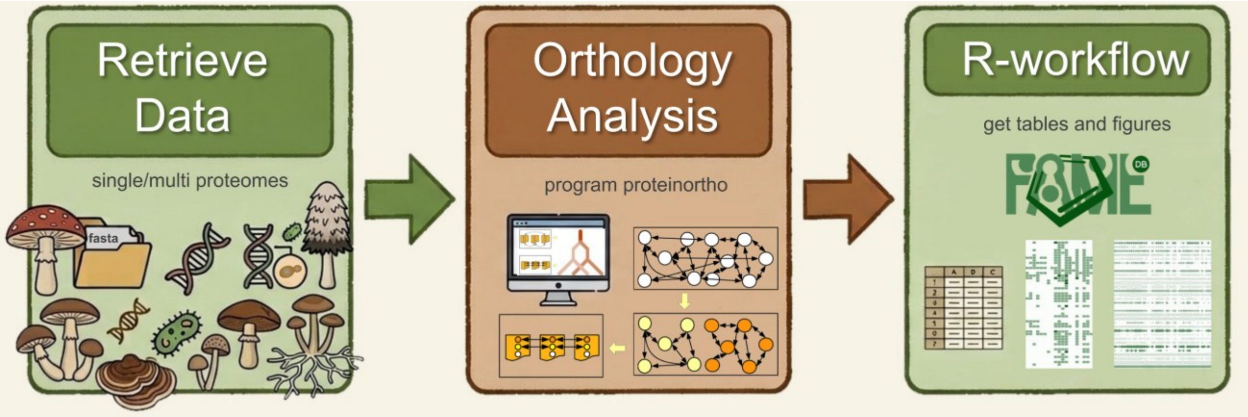

Figure S9. Schematic representation of a FAMeDB workflow.
